# Imaging Genetics in Epilepsy: Current Knowledge and New Perspectives

**DOI:** 10.3389/fnmol.2022.891621

**Published:** 2022-05-30

**Authors:** Ge Wang, Wenyue Wu, Yuchen Xu, Zhuanyi Yang, Bo Xiao, Lili Long

**Affiliations:** ^1^Department of Neurology, Xiangya Hospital, Central South University, Changsha, China; ^2^National Clinical Research Center for Geriatric Disorders, Xiangya Hospital, Central South University, Changsha, China; ^3^Clinical Research Center for Epileptic Disease of Hunan Province, Central South University, Changsha, China; ^4^Department of Neurology, The Second Affiliated Hospital of Nanchang University, Jiangxi, China; ^5^Department of Neurology, The First Affiliated Hospital of Wenzhou Medical University, Wenzhou, China; ^6^Department of Neurosurgery, Xiangya Hospital, Central South University, Changsha, China

**Keywords:** epilepsy, development, imaging, neurogenetics, endophenotype, imaging genetics

## Abstract

Epilepsy is a neurological network disease with genetics playing a much greater role than was previously appreciated. Unfortunately, the relationship between genetic basis and imaging phenotype is by no means simple. Imaging genetics integrates multidimensional datasets within a unified framework, providing a unique opportunity to pursue a global vision for epilepsy. This review delineates the current knowledge of underlying genetic mechanisms for brain networks in different epilepsy syndromes, particularly from a neural developmental perspective. Further, endophenotypes and their potential value are discussed. Finally, we highlight current challenges and provide perspectives for the future development of imaging genetics in epilepsy.

## Introduction

Epilepsy is one of the most common serious neurological disorders, affecting nearly 70 million people worldwide and adversely influencing social, physical, and psychological functions (The Lancet, 2019). In 2017, the International League Against Epilepsy (ILAE) updated the classification of seizures and epilepsy (Scheffer et al., 2017). The new classification includes etiology, age, and increasing recognition of the comorbidities, emphasizing the genetic factors on brain development and the consideration of comorbidities at each stage for precision medicine. The genetic basis of epilepsy is very complex, which comprises rare Mendelian subtypes and a likely oligo- or polygenic architecture with rare-, common- and copy number variants for more common forms (Thakran et al., 2020). Evidence in molecular genetics, animal models, and human neuroimaging supported the genetic influence on the variation of brain networks and fueled the hypothesis that mutations might affect the long-term brain maturation as early as neurodevelopment, leading to altered brain structure and function, increased seizure susceptibility, and finally, the occurrence of corresponding epilepsy and comorbidity in specific temporal points. However, known mutations only explain a fraction of patients, and the genetic risk for imaging alteration remains unclear. The main challenges include: (1) the low positive rate and reproducibility for gene identification; (2) the blurred boundary of disease classification; (3) the difficulty of coordinating multidimensional datasets during brain development defects. The emerging research field, imaging genetics, might contribute to our understanding of the biological mechanism behind epilepsy syndromes.

Imaging genetics is an integrated method that links genetic and epigenetic variations to neuroimaging measures and enables the assessment of quantitative genetic mechanisms (Arnatkeviciute et al., 2021). Currently, large cohort studies such as Enhancing Neuro Imaging Genetics through Meta-Analysis (ENIGMA) Consortium and UK Biobank, showing advantages for testing reproducibility and robustness of findings, provide an excellent platform to identify genetic underpinnings of imaging phenotypes (Hofer et al., 2020; Thompson et al., 2020). Imaging genetics has been applied in neuropsychiatry areas and is beginning to provide insight into epilepsy fields. This manuscript will review the current knowledge about genetic influence on brain structure and function, mainly in developmental and epileptic encephalopathy, genetic generalized epilepsy, and focal epilepsy syndromes, from a neural development perspective. We will explain what is known about the cellular roles of epilepsy genes and describe the potential consequences of brain imaging changes. Further, we will discuss the imaging endophenotypes and their role in etiology discovery for common epilepsies. Finally, we will prospect the potential value of novel imaging genetics technologies and multicenter, multi-disciplinary cooperation in mechanism clarification, biomarker identification, and clinical appliance in epilepsy.

## Current Knowledge of Genetic Influence on Brain Network in Epilepsy Syndromes

### Developmental and Epileptic Encephalopathy

Developmental and epileptic encephalopathy (DEE) comprises a large, heterogeneous group of age-related epilepsy syndromes characterized by specific seizure types, EEG abnormalities, and developmental regression. In 2017, the ILAE suggested the term “epileptic encephalopathy”, which highlighted the relationship between epileptic encephalopathy and developmental encephalopathy. A hypothesis is that the modification caused by genetic or environmental factors or their interactions during brain development may lead to the decisive evolution of brain networks, which might be responsible for the breakdown of normal neurocognitive networks and the formation of epileptic networks.

The majority of DEE is due to genetic disorders beyond ion channel dysfunction. These mutations involve biological pathways such as DNA repair, transcriptional regulation, axon myelination, metabolite, and peroxisomal function, which are well-positioned to have a deleterious impact on the development, migration, and apoptosis of neurons and interneurons (McTague et al., 2016). Although these genes show strong ties with long-term neurodevelopmental impairments, little is known about how microscopic molecular dysfunction affects local or whole-brain networks. One potential example of gene dynamically affecting brain network and age-related disease is the abnormal brain network change during West syndrome (WS) transition to Lennox-Gastaut syndrome (LGS).

The transition occurs in around one-third of WS children with a mean duration of 5 months–8.8 years (Calvo et al., 2020). Compared with WS possessing an abnormal parietooccipital-lenticular loop, the cortical abnormalities associated with the basal ganglia are more concentrated in the anterior lateral frontal lobe in LGS (Japaridze et al., 2013; Warren et al., 2019). Since cortical thinning is a prominent morphological feature beginning around 2 years of age, and the lateral frontal lobe likely has more thinning (Remer et al., 2017), it seems that the abnormal frontal lobe development may promote changes in the functional hierarchy and lead to a late-onset syndrome. A recent imaging genetics study from 3 to 21 years showed that accelerated cortical thinning was associated with genes related to dendritic remodeling and synaptic elimination (Ball et al., 2020). These genes partly intersect with genes involved in both WS and LGS, suggesting that the brain network alterations may result from abnormal modulation of gene expression occurring during this developmental period. However, the genetic background of DEE is heterogeneous, with several genes that can cause one syndrome, and one gene might conversely associate with phenotypic pleiotropy. Future imaging genetics research needs to decipher the precise spatiotemporal genetic modulation mechanism during this period.

### Genetic Generalized Epilepsy

Genetic generalized epilepsies (GGEs) are the most frequent type of seizures of adolescent onset, including childhood absence epilepsy, juvenile absence epilepsy, juvenile myoclonic epilepsy, epilepsy with generalized tonic-clonic seizures alone, and other genetically related generalized epilepsy. GGEs are characterized by largely polygenic inherited patterns and brain network reorganization.

#### Childhood Absence Epilepsy

Childhood absence epilepsy (CAE) typically begins between 3 and 8 years of age. The major hallmarks are typical 3 Hz spike and wave discharges (SWD) on EEG, along with brief episodes of frequent, unpredictable lapses of consciousness.

Genes associated with CAE include ligand-gated and calcium channel genes, solute carrier transporters genes, and other genes. Animal models have partly supported mutation’s effect on refining neural circuits. For example, greater dentate granule cell density and volume in the hippocampus (Richards et al., 2013), increased densities of GABA neurons, and inhibitory neuron subpopulations in the cortex have been observed in the absence of epilepsy-naive *GABAA* γ2R43Q mice (Wimmer et al., 2015), which strongly support mutation is most likely altering neural architecture in the early developmental period. Polygenic absence seizure mice also showed increased GABA neurons in ventral posterolateral thalamic nuclei (Cavdar et al., 2014). The alterations of these structures might cause deviations of normal thalamocortical circuit function and enhance susceptibility to absence seizures. For instance, McCafferty et al. (2018) discovered that top-down cortical excitation and thalamic reticular nucleus (TRN) mediated inhibition is essential for synchronous, seizure-perpetuating output of the thalamocortical neurons in the polygenic absence seizure model.

Human neuroimaging also revealed potential brain structural alterations which might be influenced by genetic factors. For example, in newly diagnosed CAE, the patients had significantly smaller frontal and temporal lobe volumes, thicker posterior cingulate and left medial occipital gyrus, and damaged white matter in the posterior corpus callosum and cingulum (Hutchinson et al., 2010; Kim et al., 2020). These differences were also found in non-newly diagnosed patients but were independent of disease duration, seizure frequency, and other epilepsy characteristics (Qiu et al., 2016; Drenthen et al., 2019). Consistent with the genetic rodent model, human EEG-fMRI elucidated the SWD production and maintenance in the thalamocortical network (Tyvaert et al., 2009; Tangwiriyasakul et al., 2018). However, these studies showed different pre-ictal cortical locations in various CAE populations (Bai et al., 2010; Moeller et al., 2010). These alterations preferentially in different cortical areas might partly relate to diverse mutations.

Clinically, the 5-year remission rate of anti-seizure medicines in CAE is 56%–65% (Camfield et al., 2014). The seizures gradually decrease to remission with age, which completely presents the whole process of the abnormal epileptic network from the development to collapse and provides an entry to study its disintegration. Therefore, longitudinal tracking of network change, particularly integrating neuroimaging and genetic alterations during the network collapse process, might provide a potentially deep understanding and new targets for CAE.

#### Juvenile Myoclonic Epilepsy

Juvenile myoclonic epilepsy (JME) develops predominantly between 12 and 18 years, characterized by myoclonic jerks and typical high amplitude diffuse and bilateral polyspike-waves on EEG. Altered cognitive dysfunctions mainly include executive and memory impairment in most cases (Iqbal et al., 2015).

To date, mutations in*GABRA1, GABRD*, *BRD2, CASR*, and gene encoding *ICK* (intestinal cell kinase) have been found in JME families (Santos et al., 2017). Functional expression studies of the *ICK* variants in progenitor cells showed impaired proliferation and mitosis, suggesting abnormal migration of cortical progenitors (Bailey et al., 2018). Using the rodent model, McCarthy et al. (2020) reported that *BRD2* gene haploinsufficiency led to GABA neuron deficits in the striatum and primary motor neocortex as early as P15, and seizure susceptibility increased from the P30 but not in younger animals (corresponds to the human JME onset pattern). Therefore, these variants are supposed to drive the abnormal functions of neural progenitor cells, induce microarchitecture changes and lead to epilepsy in specific periods through brain maturation. Nonetheless, the causality and bioinformation of these mutations require further ascertainment.

Transition issue also occurs in CAE and JME (Camfield et al., 2014). Autosomal dominant mutations in the *GABRA1* are associated with both CAE and JME. Arain et al. (2015) reported that haploinsufficient and dominant-negative mutations led to absence seizures at P35 and persisted at P120 in mutant mice. However, more frequent spontaneous and evoked polyspike-wave discharges and myoclonic seizures were observed at P120, suggesting that shared mutations might lead the disease to evolve from CAE to JME.

For neuroimaging studies of new-onset JME patients, reduced thalamic volume and abnormal white matter values were found (Ekmekci et al., 2016; Perani et al., 2018). Two prospective longitudinal experiments supported dynamic network reorganization compared to normal development, including less modular cortical network and greater frontoparietotemporal volume and thickness (Lin et al., 2014; Garcia-Ramos et al., 2018). Moreover, structural and functional imaging characteristics, especially the frontal and temporal lobes, have been identified as endophenotypes (Caciagli et al., 2019, 2020), suggesting that imaging abnormalities in patients with JME might partly be influenced by genetic factors.

From a developmental perspective, regions of the temporal and frontal lobes exhibited the greatest variations in thinning across the maturation period. Imaging genetics has suggested that cortical thinning both in late childhood and adolescence is closely tied to the expression levels of spine and dendrite gene panels, which contain several CAE and JME genes (Parker et al., 2020). In a word, the cortical and subcortical regions may follow an abnormal developmental trajectory in CAE and JME, in which genetic factors probably play a significant role. Clarifying the precise spatiotemporal modulation of gene expression profiles on imaging attributes in the future will benefit the investigation of the mechanism of seizure onset, cognitive dysfunction, and prognosis.

#### Juvenile Absence Epilepsy and Epilepsy With Generalized Tonic-Clonic Seizures

Juvenile absence epilepsy (JAE) onset occurs typically between 9 and 13 years. Mutations in ion channel genes and *INHA* may be involved in a subset of patients (Thakran et al., 2020). Network parameters showed increased node efficiency in the JAE bilateral supplementary motor area, independent of clinical variabilities (Zhang T. et al., 2021). In contrast to CAE, subtle structural alterations in JAE are mainly in frontal nodes but not in the thalamus (Tondelli et al., 2016). The brain network differences might relate to environmental and genetic factors and associate with different medication responses in different epilepsy syndromes.

Epilepsy with generalized tonic-clonic seizures (EGTCS) commences in teens and young adults with generalized tonic-clonic seizures that occur primarily within 2 h after awakening. The genetic architecture of EGTCS is complex, and no genetic variants are found in most cases. Gray matter reductions were found most pronounced in the medial frontal gyrus and the ventral thalamic nuclei, which showed no correlation with the clinical characteristics (Ciumas and Savic, 2006). However, although JAE and EGTCS are common phenotypes of GGE, the role of genetics is still highly elusive.

Compared to CAE with two-thirds remit at adolescence, a high proportion of adolescent-onset GGE patients require lifelong treatment because withdrawal could cause a relapse even in people who have been seizure-free for many years with appropriate drugs (Vorderwülbecke et al., 2017). The different disease processes might relate to changed neural plasticity and compensatory capacity during brain development (Helmstaedter and Witt, 2012). The collapse of the normal brain network and an insufficient recovery or compensation might occur in adolescent-onset GGEs. However, these abnormalities in brain networks that are associated with seizures and comorbid behavioral changes might be influenced by various environmental and genetic factors. Therefore, prospective studies comparing the mutation-related abnormal network in subtypes of drug-naive GGEs will help distinguish the pathogenesis and benefit precise treatment.

#### Benign Adult Familial Myoclonic Epilepsy

Benign adult familial myoclonic epilepsy (BAFME), also known as familial cortical myoclonic tremor with epilepsy and autosomal dominant cortical myoclonus and epilepsy, is a syndrome with clinical and genetic heterogeneity and is not yet officially recognized. Pentanucleotide expansions of TTTCA and TTTTA in different genes (BAFME 1: *SAMD12*, BAFME 2: *STARD7*, BAFME 3: *MARCH6*, BAFME4: *YEATS2*) have been recognized to play an essential role in its pathogenesis (Latorre et al., 2020). Although exact mechanisms remain unclear, non-coding repeat expansions irrespective of the genes revealed that RNA toxicity is the candidate pathomechanism (Cen et al., 2018; Ishiura et al., 2018). These genes are highly expressed in the cerebellum and can cause related local imaging alterations. Multimodal imaging studies have investigated abnormal metabolite patterns, local fluctuation, and atrophy in the BAFME cerebellum (Striano et al., 2009; Buijink et al., 2013, 2016; Long et al., 2015; Wang et al., 2020).

However, BAFME is clinically characterized by cortical tremor and myoclonus with giant somatosensory-evoked potential and long-latency cortical reflex, suggesting that the movements are generated by abnormal sensorimotor discharges (Latorre et al., 2020). The inconsistency between cortical hyperexcitability and cerebellum pathology raises the hypothesis that abnormal cerebellar inputs may induce the disinhibition of cortical neurons and cause cortical tremors/myoclonus in BAFME. This hypothesis was further confirmed by imaging studies. Long et al. (2016) observed altered cerebellar-cerebral functional connectivity in the default network, dorsal attention network, and control network. Wang et al. (2020) showed increased functional connectivity between the cerebellum and the precentral gyrus. The cortical dysfunctions and altered cerebellar-cerebral loop suggest that the main disease mechanism is a network impairment and highlight that noncoding-repeat expansions may cause the disruption of the neural network and induce cortical seizures in BAFME (Latorre et al., 2020).

### Focal Epilepsy Syndromes

Several focal epilepsies with genetic components have been identified. Mostly rare familial epilepsy syndrome displaying Mendelian inheritance. Moreover, the genetic basis of familial focal epilepsy might also relate to common sporadic focal epilepsies. Research of molecular function, animal models, and neuroimaging preliminarily expose those mutations contributing to abnormal brain development and epileptogenesis.

#### Sleep-Related Hyper Motor Epilepsy

Sleep-related hyper motor epilepsy (SHE), previously known as nocturnal frontal lobe epilepsy, is a heterogeneous syndrome with acquired injuries and genetic causes. The autosomal dominant pattern of SHE (ADSHE) has been associated with mutations in several genes, including genes encoding nAChR (mainly in *CHRNA4*, *CHRNB2*, *CHRNA2*), *DEPDC5*, *NPRL2*, *NPRL3, KCNT1*, and *CRH* (Perucca et al., 2020). In addition, these mutations have also been reported in some sporadic cases (Chen et al., 2009; Liu et al., 2011).

Approximately 12% of the pedigrees carry mutations in genes encoding nAChR. Fukuyama and Okada (2020) revealed the age-dependent and event-related epileptogenesis in the S286L-mutant *CHRNA4* mice model (corresponding to the human S284L-mutant *CHRNA4* gene). The mutant impairs the activation of GABAergic transmission from the TRN to thalamic nuclei, weakening Erk signaling inhibition and gently increasing connexin 43 expression and glutamatergic transmission in the thalamocortical pathways. At the critical seizure onset period, factors (such as physiological sleep spindle and pathological interictal discharges) cause persistent hemichannel opening and excitatory neurotransmitter release, leading to the disruption of several homeostasis systems and orbitofrontal epileptic seizures.

In ADSHE patients with an identified nAChR gene mutation, Picard et al. (2006) found decreased nAChR density in patients’ prefrontal cortex, which explained why mutations in receptors presented in the entire brain result in focal frontal lobe epilepsy. Fedi et al. (2008) also showed reduced D1 receptor binding at the striatum, suggesting that mutation might modify a wide range of other functions. In more common sporadic SHE patients, metabolic alterations were observed in the dorsolateral prefrontal cortex (Wang W. et al., 2021). Although the pathomechanism is partially well revealed in the *CHRNA4* mice model, many unsolved challenges exist. For instance, what is the neuroimaging foundation for patients with different variants? The mechanism is essential since patients with various variants in the nAChR gene showed good or poor drug responses (Fukuyama and Okada, 2020). Moreover, genetic underlying in sporadic patients is largely unclear, suggesting neural mechanisms are complicated and need detailed exploration.

#### Familial Focal Epilepsy With Variable Foci

Familial focal epilepsy with variable foci (FFEVF) is a focal seizure that begins at any time from infancy to adulthood. The variable foci mean that seizures emanate from various cortical locations in different family members. However, for each individual, a single focus remains constant throughout their lifetime (Picard et al., 2000). FFEVF is the paradigmatic phenotype of GATOR1 related Mendelian focal epilepsy, which has mutations in the GATOR1 protein complex (*DEPDC5*, *NPRL3*, and *NPRL2*, Weckhuysen et al., 2016). GATOR1 protein complex plays a pivotal role in regulating mTOR signaling and several downstream effects in brain development, such as proliferation, differentiation, migration, dendrite formation, and plasticity. Animal model and resected human brain tissue suggest mutations in *DEPDC5, NPRL3*, and *NPRL2* increase mTOR pathway activation, which could disrupt neuronal morphogenesis and cortical lamination, increase neuronal excitability, and might be implicated in epileptogenesis by altering the formation of neural circuits (Weckhuysen et al., 2016; Hsieh et al., 2020; Klofas et al., 2020).

Detailed brain imaging indicated that a subset of patients with GATOR1 mutations had structural brain abnormalities, particularly focal cortical dysplasia (Weckhuysen et al., 2016; Baldassari et al., 2019). The mTOR pathway had been thought to play a significant role in focal cortical dysplasia as a ubiquitous regulator. One interesting question is that instead of causing mTOR hyperactivation in the whole brain neurons, heterozygous germline mutation normally leads to local mTOR activation and cortical dysplasia. Animal model studies suggested that biallelic inactivation was necessary to cause mTOR hyperactivation, shape neuronal morphology, and generate epilepsy with focal cortical dysplasia (Ribierre et al., 2018). Resected human tissue specimens demonstrated somatic mutations plus the germline mutation caused a functionally homozygous condition (Baldassari et al., 2019). The somatic mutational load was higher in the epileptogenic zone with increased concentrations of dysplastic cells, whereas lower or absent in the surrounding areas. These studies provide strong evidence that the somatic “second hit” mutations in cortical neurons rather than heterozygous germline mutation alone cause mTOR signaling hyperactivation and focal abnormal neurodevelopment.

Another clinical challenge is the phenotypic heterogeneity related to mutations in the mTOR signaling pathway. For example, mutations in GATOR1 could induce a range of epilepsy syndromes. In addition to FFEVF, other Mendelian (ADSHE, familial mesial temporal lobe epilepsy) and sporadic focal epilepsies have been reported (Baldassari et al., 2019). Mutations in other mTOR pathway genes, such as *TSC1, TSC2, AKT3, MTOR*, and *PIK3CA*, are linked to some epilepsy-related multisystem disorders (e.g., tuberous sclerosis complex; Nguyen and Bordey, 2021). The “second hit” mechanism might involve region- and age-specific effects of the mTOR pathway genes, which provide a plausible explanation for the wide range of phenotypic variability (Rivière et al., 2012; Ribierre et al., 2018; Goswami and Hsieh, 2019). Further combining genetics and neuroimaging would be explorable to decipher these spatiotemporal effects.

#### Familial Temporal Lobe Epilepsy

Autosomal dominant lateral temporal lobe epilepsy (ADLTE), also known as autosomal dominant epilepsy with auditory features, displays autosomal dominant inheritance with reduced penetrance. The most common gene is *LGI1*, of which variants account for around 30% of families (Michelucci et al., 2013). *RELN* and *MICAL-1* have been recently identified as susceptibility genes, which together may account for another 30% of pedigrees (Dazzo et al., 2015, 2018). Variants in *LGI1* are related to altered synaptic function and neuronal morphology in the hippocampus (Zhou et al., 2009; Hivert et al., 2019). Reelin (encoded by *RELN*) co-localizes with LGI1 in the temporal and hippocampus, suggesting a convergent signaling pathway (Michelucci et al., 2017). In addition, the *MICAL-1* variants cause dysregulation of the actin cytoskeleton dynamics and could also serve abnormal cell maturation and migration (Dazzo et al., 2018). Therefore, MRI of patients carrying mutations could be expected to show even minor changes. Indeed, dysplastic features and abnormal white matter values in the temporal gyrus were found in patients with genetic mutations (Tessa et al., 2007).

Familial mesial temporal lobe epilepsy (FMTLE) is a heterogeneous syndrome with unclear genetic architecture. Although mutations in mTOR pathway genes have been identified, they might not be the frequent cause of FMTLE (Striano et al., 2015; Wang Y. et al., 2021). The most common neuroimaging findings of FMTLE are HS and focal cortical dysplasia (Dührsen et al., 2018), but whether HS is affected by genetic factors remains unclear. MRI studies for both FMTLE patients and their asymptomatic siblings showed similar but mild HS in siblings (Alhusaini et al., 2016). However, no genes have yet been strongly associated with this imaging feature.

#### Childhood Epilepsy With Centro Temporal Spikes

Childhood epilepsy with centrotemporal spikes (CECT), previously known as benign childhood epilepsy with centrotemporal spikes or rolandic epilepsy, is a common focal syndrome with complex inheritance patterns. The most relevant gene for CECT is GRIN2A. Other related genes include ELP4, BDNF, KCNQ2, KCNQ3, RBFOX1, and DEPDC5 but require further investigation (Xiong and Zhou, 2017). *GRIN2A* encodes the GluN2A subunit of the NMDA receptor and transmits signals involved in brain development and synaptic plasticity (Mota Vieira et al., 2020). Recently, neuroimaging has ascertained the developmental delay in CECT, which is associated with neuroanatomic changes in the Rolandic regions (Zhang Q. et al., 2021). These results suggest that CECT is related to abnormal brain development during childhood.

Moreover, the GRIN2A-related epilepsy-aphasia spectrum is broad and ranges from the mild end, such as CECT, to the severe end, such as Landau-Kleffner Syndrome (LKS) and Continuous Spike and Wave During Sleep (CSWS) in DEE (Carvill et al., 2013; McTague et al., 2016). Recent studies have suggested that the clinical heterogeneity might relate to diverse mutations in *GRIN2A* (Strehlow et al., 2019). Nonetheless, there are still many unclear mechanisms. For example, what are the neuroimaging alterations during the developmental period? How do these variants lead to different brain changes and epilepsy-aphasia phenotypes? What is the genetic basis of patients without the *GRIN2A* mutations?

Collectively, genetic factors may be involved in the development of some abnormalities in focal epilepsies. For patients with a relatively clear cause, gene mutations affect one or combined developmental steps, including neuronal maturation, proliferation, migration, and layer organization, and might lead to seizures in various periods. However, when and where the brain abnormalities emerge and where changes in the brain fit on the pathway from molecular dysfunction need further investigation. It is essential since early intervention before patients present with symptoms might reverse neuronal abnormalities and seizures (Zhang et al., 2020; Nguyen et al., 2021). most sporadic focal epilepsies, the underlying mechanisms are unclear. Association analysis with whole-genome sequencing to search for causative common and rare variants might reveal the genetic basis of epilepsy more effectively.

## Imaging Endophenotype in Common Epilepsy

The genetic determinants of common epilepsy syndromes are much less clearly defined. The ILAE Consortium on Complex Epilepsies reported the largest genome-wide mega-analysis from 15,212 epilepsy cases and 29,677 controls, revealing 16 genome-wide significant loci (Epilepsies, 2018). Despite these advances, the number of novel genome-wide significant findings and functional validation remains sparse. Intermediate quantitative trait (endophenotype), a biological measure closer to disease molecular causes than clinical assessments, is a possible solution for deciphering the effect of pathogenic variants on brain structure and function. Neuroimaging characteristics are quantitative traits (QT) of brain structure or function. Imaging QTs show strong tissue specificity and can more greatly index genetic liability for an illness. For example, the brain white matter heritability estimated by central nervous system inactive SNPs was significantly smaller than active SNPs (Zhao B. et al., 2021). The use of quantitative imaging endophenotypes in genomic studies is proposed to reduce phenotypic heterogeneity, boost statistical power, and enhance biological interpretation (Rose and Donohoe, 2013; Xu et al., 2017). So far, several imaging-based studies have attempted to explore neuroimaging endophenotypes in epilepsy patients and their asymptomatic first-degree relatives.

In recent years, substantial progress has been made in the imaging endophenotype of GGEs. JME patients and their asymptomatic relatives appear to exhibit similar brain structural and functional alteration patterns, including increased folding complexity and surface area in prefrontal and cingulate cortices (Wandschneider et al., 2019), abnormal volume and morphological characteristics of the hippocampus (Caciagli et al., 2019), and increased motor system and hippocampus activation (Caciagli et al., 2019, 2020). These regions also displayed increased geodesic distance, suggesting network isolation and decreased efficiency (Wandschneider et al., 2019). Similarly, in GGE populations, elevated sensorimotor network synchrony (Tangwiriyasakul et al., 2019) and interictal cortical power and connectivity (Stier et al., 2021) have been reported in patients and their asymptomatic siblings. These subtle but similar patterns in asymptomatic siblings suggest that genetic mechanisms might play a role in the abnormal development of thalamofrontal brain networks. Nonetheless, the specificity of frontotemporal endophenotypic property for a specific disease needs further proof.

For temporal lobe epilepsy, hippocampus volume appears to be the most promising neuroimaging endophenotype, particularly in MTLE with a strong family history of seizures (Alhusaini et al., 2016). However, this endophenotype in sporadic MTLE remains controversial. Alhusaini et al. (2019) and Yaakub et al. (2019) used automated segmentation tools and reported no significant hippocampus volume deficits in the asymptomatic siblings. While using a manually segmented method, Long et al. (2020) observed smaller bilateral hippocampal volumes and subregional atrophy in the right hippocampus in TLE patients and siblings. Possible explanations for this controversial consequence might relate to the limited sensitivity of the automated segmentation technique. In addition, genome-wide association studies (GWAS) have identified common variants for hippocampus volume in other populations (Hibar et al., 2017), highlighting the need for large samples to replicate previous findings in TLE. Other structural alterations beyond the hippocampus, such as the morphology of the ipsilateral temporal lobe, white matter microstructure, and EEG features of alpha oscillations were also noted in the asymptomatic siblings of MTLE (Alhusaini et al., 2016; Yaakub et al., 2020). These disease-associated imaging endophenotypes are important to potentially index genetic liability and contribute to understanding gene function in common epilepsies.

Characterizing neuroimaging endophenotypes in common epilepsy represents the first step toward imaging genetics. Screening the whole genome for neuroimaging derived endophenotypes will undoubtedly provide valuable insight. As imaging genomics collaborations, the discovery rate continues to increase, and endophenotypes have been successively used for genetic exploration in neuropsychiatric diseases (Meyers et al., 2017). However, imaging endophenotypes have yet to be applied in epilepsy imaging genetics study, and some attention should be paid when adopting this approach. The main criticisms of imaging endophenotypes include their unclear genetic basis and single disease-specific. Currently, the genetic variations of epilepsy imaging endophenotypes are unclear. It seems that genetic environment interaction contributes to some endophenotypes. For example, early environmental effects such as brain injury, infection, and febrile seizure history are known risk factors for hippocampus atrophy in TLE patients with HS. Although genetic factors seem not to affect the epilepsy risk directly, they operate by increasing susceptibility to the consequences of early brain insults (Perucca and Scheffer, 2021). Endophenotype Ranking Value (ERV) represents an unbiased and empirically derived statistic for selecting optimal endophenotypes (Glahn et al., 2012). It is defined as the product of the square root of the heritability of the illness, the square root of the heritability of the endophenotype, and their genetic correlation. ERV has been applied to psychosis and cognitive ability to identify a set of objective endophenotypes, where a higher value indicates that the candidate endophenotype and the illness are more strongly influenced by shared genetic factors (Knowles et al., 2021). Therefore, further studies can conduct ERV to estimate the proportion of shared genetic factors influencing both the endophenotype, such as imaging phenotypes, and the clinical endpoint, such as epilepsy. Another important point to consider is the high burden of comorbidities in epilepsy. For example, frontal and temporal lobe alterations also serve as endophenotypes in other psychiatric disorders such as autism and bipolar disorder (Spencer et al., 2012; Choi et al., 2020). Shared genetic mechanisms between epilepsy and other psychiatric disorders mean that endophenotypes can not necessarily be disorder-specific (Peng et al., 2021). Further work on discovering and confirming transdiagnostic endophenotypes in large cohorts and exploring the mutations from a genome-transcriptome-endophenotype-phenotype-validation perspective might effectively address the entirety of the disease, i.e., seizures and comorbid conditions.

## Shared Imaging and Genetics in Epilepsy and Comorbidity

The above epilepsy syndromes are often present with comorbidities. Several studies have observed cognitive and behavioral abnormalities in both children (Austin et al., 2001; Hermann et al., 2012) and adults (Taylor et al., 2010; Witt and Helmstaedter, 2015) at or before epilepsy onset. Therefore, besides clinical factors such as disease duration, seizure type and frequency, and medications, other potential etiologies, including genetic etiologies, need detailed exploration.

In epilepsy syndromes with relatively clear genetic causes, several genes have also been discovered as solid biomarkers of psychiatric conditions, such as cognitive disorders, autism, attention-deficit hyperactivity disorder, and migraine (Noebels, 2015; McTague et al., 2016). In common epilepsy with complex etiology, studies have identified epileptic variations associations with comorbidities, including memory impairment, executive dysfunction, working memory impairment, slowed processing speed, depression, and anxiety (Hermann et al., 2021). In addition, cognitive and behavioral endophenotypes have been observed in unaffected siblings of patients with various epilepsy syndromes, including impaired prospective memory, psychomotor speed, phonemic verbal fluency, and executive function in JME, altered reading, language, attention in CECT, and abnormal visuospatial function in TLE (Wandschneider et al., 2010; Smith et al., 2012; Verrotti et al., 2013; Chowdhury et al., 2014; Iqbal et al., 2015; Tan et al., 2020). Moreover, a recent study has revealed that TLE-associated memory status may be influenced by different expressions of genes in pathways pertaining to neurological dysfunction and neurodegenerative diseases (Busch et al., 2020). These results suggest the potential genetic contributions to the comorbidities of epilepsy, which might relate to shared imaging endophenotypes, such as hippocampal volume and frontal cortical morphology (Reitz and Mayeux, 2009; Shaw et al., 2015). Therefore, future identification of genomic risk for comorbidities in epilepsy and related brain changes is essential for precise diagnosis and treatment.

## Challenges and Future Directions of Imaging Genetics Methods in Epilepsy

**Figure 1:**
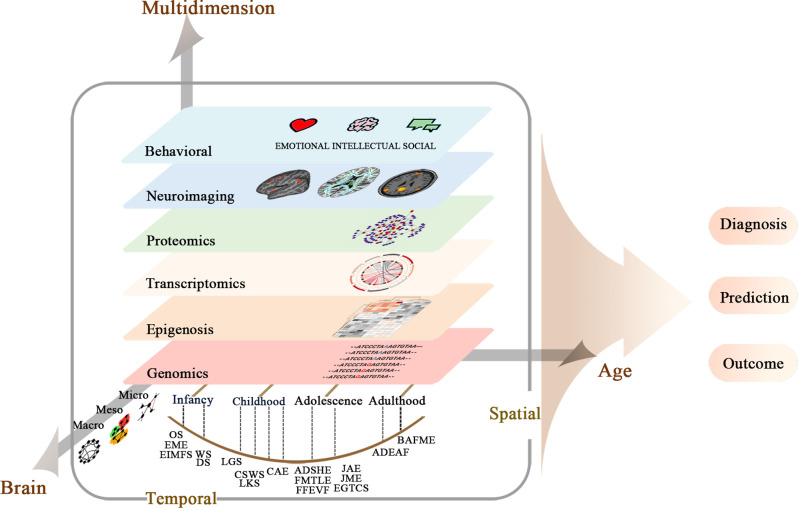
Multi-scale and multi-dimensional approaches for system understanding of neural mechanisms of epilepsy syndromes.

According to current genetic and imaging knowledge, there is a growing awareness of the close link between epilepsy mutations and brain alterations during brain development. However, the research on the neural mechanism mainly focuses on basic research and pathophysiology, and there is still a considerable gap in guiding clinical diagnosis and treatment. Future imaging genetics will reveal comprehensive mechanisms and identify imaging genetic biomarkers for epilepsies and comorbidities. For DEE and Mendelian genetic epilepsy, prospective imaging genetics studies with homogeneous populations and multi-sources data integration are informative in clinical practices. For common epilepsy syndromes, major challenges, including lack of a definitive molecular diagnosis, complicated epigenomic modification, modest sample sizes, and lack of integration, vigorously encourage the field to grow in emerging imaging genetics directions (Figure 1).

### New Technologies of Imaging Genetics Applied for Mechanism Clarification

The advances in imaging and genetic technologies, including capturing finer brain imagings through texture or voxelwise analysis and adopting inexpensive sequencing and genotyping methods for GWAS, have been particularly crucial in modern genetic imaging analysis. In fact, GWAS imaging methods have evolved from SNP-targeted QT analysis to SNP-multiple QT analysis across the entire brain and genome. Attributed to large-scale studies, associations for a range of imaging attributes in normal and neuropsychiatric populations have been detected (Hofer et al., 2020; Zhao B. et al., 2021). However, some major challenges, including multiple testing penalties at the cost of reduced statistical power, modest SNP effect sizes, and unclear biological implications, remain to be overcome. Novel imaging genetics technologies destined to solve the heavy burden of comparison and gain meaningful insights will benefit in identifying new candidate variants and comprehending complex associations.

#### *Post-hoc* GWAS Analysis for Loci Identification and Biological Interpretation

*Post-hoc* GWAS have recently emerged that permit integration of functional genomic annotations with GWAS summary statistics and translation into explicable biological mechanisms and models.

##### *Post-hoc* Quantitative Trait Loci Analysis

Most of the GWAS signals map to non-coding regions, which potentially contribute to complex disease phenotypes by regulating QTs, such as gene expression and neuroimaging. The genetic loci contributing to a QT are called quantitative trait loci (QTL), which may act as endophenotypes between disease variation and disease phenotype (Neumeyer et al., 2020). Combined with GWAS, QTL studies afford greater power to detect associations and interpret the contributions of disease-associated variants. At present, a number of different QTLs with potential genetically regulated components have been investigated, ranging from molecular levels, such as gene expression (eQTL), DNA methylation, histone modifications, and chromatin accessibility, to higher levels of intermediate phenotypes, such as metabolites and neuroimaging. Of these, eQTLs are the most commonly used, and several kinds of methods have been developed to integrate GWAS results and eQTL analysis.

One approach is based on gene expression imputation, such as the transcriptome-wide association study (TWAS; Wainberg et al., 2019). This method estimates the genetically regulated component of expression using reference transcriptome datasets and tests that component for association with disease. Another method is based on the colocalization of eQTLs and GWAS variants, which suggests that the eQTL target gene could be involved in the molecular pathway underlying the complex disease (Hormozdiari et al., 2016). In addition, eQTLs can serve as instrumental variables in Mendelian Randomization (MR) studies for mapping the causal path from genetic variants to disease (Neumeyer et al., 2020). These methods can be extended to integrate imaging GWAS results and other QTL analyses to elucidate distinct biological processes in brain alterations (Zhang et al., 2019).

Moreover, imaging genetics can integrate GWAS between genetic regions and diseases with imaging QTL. Similar to TWAS, Imaging wide association study (IWAS) reduces the burden of tests by considering the association between genetically regulated components of imaging endophenotype and clinical phenotype (Xu et al., 2017). In addition, the colocalization approach will pinpoint shared causal variants impacting both brain character and risk for disorders, and MR can test whether the effect of a GWAS SNP on a specific trait is mediated by imaging endophenotypes (Knutson et al., 2020).

Several issues should be highlighted in the QTL analysis. First, it is difficult to find the related genes if the most relevant disease-, tissue-, cell-, and developmental-specific QTL are not available. Second, each QTL is only one dimension of genetic regulation underlying diseases. Third, the statistical power is increased only when the genetic component of the trait has a causal effect on the endophenotype. Recently, several methods, in addition to ERV, can be used to identify causal endophenotypes. For example, MR and univariate-IWAS identify endophenotypes with the assumption that the SNPs only affect disease by means of the endophenotype being tested (Xu et al., 2017; Knutson et al., 2020). In addition, the multivariate-IWAS model provides causal effect estimation by accounting for multiple potential intermediate pathways (Knutson et al., 2020). Furthermore, context-specific QTL maps for comprehensive characterization and downstream interpretation of disease-associated variants have been used in Alzheimer’s disease (Patel et al., 2021). Future identifying context-specific QTL in epilepsy and integrating multi-dimensions of biological information can prompt the understanding of the mechanisms underlying epilepsy.

##### *Post-hoc* Enrichment Analysis

Enrichment analysis is routinely used to link highly polygenic signals based on GWAS to a limited number of biological processes, including cell types, biological pathways, or developmental stages. Brain imaging genetics studies usually apply the standard enrichment methods used in the genomic domain (threshold-based and rank-based approaches). However, novel methods leveraging prior imaging and/or genetic knowledge and considering the collective effect of SNP/gene sets and/or QT sets have been proposed specifically for brain imaging genetics. These methods might outperform traditional enrichment approaches and are able to identify a number of new significant associations. For example, similar to traditional enrichment analysis treating a pathway as a set of SNPs or genes, Regional Imaging Genetic Enrichment Analysis treats the imaging regions of interest as a set of voxels. A *post-hoc* enrichment analysis was performed on the voxelwise GWAS statistics to identify regions of interest over-represented by the top voxel findings (Yao et al., 2020). In addition, two-dimensional Imaging Genetic Enrichment Analysis jointly considers meaningful gene sets and brain circuits based on brain transcriptome data, and aims to identify gene-imaging QT pairs over-represented by SNP-imaging QT findings from brain-wide genome-wide imaging genetic association study (Yao et al., 2017).

#### Multivariate Analysis for High-Level Association Identification

Imaging genetics also adopts multivariate techniques to mine the complex association between genes and quantitative imaging traits (e.g., epistatic effect, phenotypic pleiotropy, and genetic heterogeneity; Shen and Thompson, 2020). Several data-driven multivariate approaches have been proposed for imaging genetics, such as sparse reduced rank regression (sRRR), sparse canonical correlation analysis (sCCA), and parallel independent component analysis (pICA). These methods are designed to extract latent variables from both genetic and imaging data and maximize the links between latent genotype-phenotype pairs. For example, sRRR minimizes the error of the multivariate regression by reducing the rank of the regression matrix. sCCA maximizes the correlation between two modalities, while pICA extracts maximally independent components from each modality as well as simultaneously optimizes the correlation. Since multimodal imaging data may carry complementary information, novel multimodal sCCA models have been proposed to combine multimodal imaging data with genetic data (Du et al., 2020). In addition, extensions of multivariate analysis, which incorporate prior knowledge such as genes, pathways, and known trait-associated variants, can simplify model complexity and offer meaningful biological insights for data-driven estimation (Bi et al., 2020).

#### Longitudinal Imaging Genetics Study for Progressive Brain-Changing Mechanisms

It is now appreciated that epilepsy is highly heterogeneous at the phenotypic and genomic levels and that they continue to evolve over time. Thus, the epilepsy field requires not only *in vitro* imaging genetic biomarkers but also spatially and temporally resolved biomarkers. The value of neuroimaging genetics in epilepsy stems from the fact that patients can acquire multiple times imaging during various developmental and disease stages, thus making it possible to understand the roles of genetic factors on progressive brain changes. Several models have incorporated longitudinal imaging data into the imaging genetic framework. For example, temporal multi-task sCCA models (T-MTSCCA) make full use of the complementary information carried by imaging QTs at different time points through learning a canonical weight matrix for SNPs, where each column corresponds to one SCCA task between each longitudinal imaging modality and SNPs. In addition, T-MTSCCA maximizes the canonical correlation for each time point separately while do not require the genetic component to maintain the same across all the time points. This means this method takes brain heterogeneous progressive patterns (different SNPs at different time points may contribute to regional variations) and longitudinally correlated SCCA tasks into account and is able to identify a trajectory of progressive and time-dependent imaging genetic patterns (Du et al., 2019). Future integration of longitudinal transcriptome and epigenome data in statistical models might help uncover the dynamic interaction pattern from imaging and genomic levels and offer biomarkers for disease progression.

#### Transcriptome-Imaging Analysis for Spatiotemporal Regulatory Mechanisms

Brain-wide gene-expression atlases open opportunities to investigate the mechanisms between gene expression and brain variations. Allen Human Brain Atlas offers an anatomically comprehensive expression atlas (more than 20,000 genes in 3,702 regions; Hawrylycz et al., 2012). By comparing a spatial imaging phenotype (quantifies age-associated changes or case-control differences) to the spatial expression pattern of each gene, transcriptomic imaging studies can shed light on the molecular correlates of brain changes in both normal and aberrant neurodevelopment. In addition, detailed solution for challenges in transcriptomic imaging data (e.g., intrinsic spatial autocorrelation, specificity of identified associations, false-positive bias in enrichment) enables more robust and rigorous inference (Fulcher et al., 2021; Markello and Misic, 2021). As the field develops, spatiotemporal transcriptome and other functional genomics atlases covering the entire human lifespan will provide molecular correlates of disease-related brain changes from a developmental perspective (Wang et al., 2018; Eze et al., 2021; Song et al., 2021). In addition to human-specific atlases, detailed brain spatiotemporal expression atlas can be measured in animal models such as mice, for which specimens are more readily available (Han et al., 2018). Along with dual *in utero* electroporation tools, studies can even manipulate the gene expression in a spatially and temporally regulated manner (Zhang et al., 2022). Thus, these techniques allow for mutation discovery and their region- and age-specific effects investigation.

Collectivity, the rapid growth of statistical and machine learning methods offer the possibility of studying epilepsy imaging genetic mechanisms at multiple spatial and temporal scales, which can effectively identify both common and rare genetic variants related to imaging and ultimately help complete the causal line of genome-transcriptome-endophenotype-phenotype.

### New Technologies of Imaging Genetics Applied for Clinical Appliance

Imaging genetics also lies their power in multi-source data integration to comprehensively reveal the neural mechanism of epilepsy, thereby discovering new targets to assist diagnosis and prediction. Previous studies that used methods such as support vector machines, neural network analyses, ontology-based and genetics-based data mining algorithms have demonstrated that molecular genetics, electrophysiology, and brain structure and function data can be effectively applied to the classification and diagnosis of epilepsy. Moreover, machine learning has made progress in seizure prediction and prognosis assessment (Abbasi and Goldenholz, 2019). These studies indicate that multi-source heterogeneous data can reveal the pathogenesis of epilepsy from different levels. However, most of the current analyses extracted the feature from a single modality or simply concatenated the feature vectors from different modalities, which means they did not make full use of mutual information and hierarchical relationship between different modalities to comprehensively and objectively establish the neural mechanism model. Multi-view learning, also known as data integration from multiple feature sets, is a rapidly developing direction in machine learning. This approach can exploit the complementarity of heterogeneous data (e.g., genome, transcriptome, epigenetics, proteome, neuroimaging, electrophysiology, and environment) to improve model performance and have great potential in realistic clinical scenarios. For example, semi-supervised classification theory can handle both disease label insufficiency and label inaccuracy (Huang et al., 2021). In addition, the view-consistency method can deal with incomplete multi-view data collected from diverse domains (Yang et al., 2022). In the foreseeable future, robust multi-view learning theories and methods that are more oriented to clinical scenarios will be of great significance for moving beyond the current dichotomous, improving disease prediction in the early lifetime, and advancing clinical management.

### Multicenter Cooperation for Epilepsy Imaging Genetics

Future imaging genetic research in epilepsy will need to be enhanced by multicenter cooperation and multidimensional datasets. The ENIGMA-Epilepsy working group is one such effort aggregating brain imaging, clinical, and genome-wide genetic data from various epilepsy populations and beginning to evaluate the effect of genes on brain measures (Sisodiya et al., 2020). Further including other ethnic populations and building epilepsy reference multidimensional imaging genetic datasets will help explore the complex interplay between the genome, brain, behavior, and environment, and ultimately benefit the clinical applications.

In addition, large brain projects (e.g., UK Brain Expression Consortium, Genotype-Tissue Expression Project, BrainSeq, BrainSpan, and BrainCloud) using post-autopsy brain data have shed light on the neural mechanism in many psychiatric disorders (Wang et al., 2019). Although the number of brain samples in epilepsy is relatively limited and understudied yet (Hernandez-Ronquillo et al., 2020), surgical tissues (e.g., malformation, hippocampus sclerosis) provide a unique source for genetic information. Furthermore, rapid developments in single-cell and spatial transcriptomics enable consideration of cell type or cell state and associated spatial locations (Longo et al., 2021). Therefore, the future establishment of the epilepsy brain bank will facilitate the comprehensive genetic regulatory profiling and accurate spatiotemporal imaging genetics of the epilepsy brain at different scales.

### Multi-disciplinary Team in Future Epilepsy Research

Given the unprecedented scale and complexity of the brain imaging genomics datasets, it is increasingly evident that future progress in epilepsy will require a team effort. The combination of molecular geneticists, bioinformaticians, developmental biology, computer science, and mathematical modeling will offer a powerful framework for linking microscale models of epilepsy pathophysiology with macroscale measures of brain dysfunction in various developmental periods. The goal of this review is to introduce the potential of imaging genetics in epilepsy to a wide audience of neurologists, geneticists, neuroscientists, radiologists, and engineers. Although imaging genetics has already entered its 3rd decade, this method is scarcely applied to epilepsy. Since many challenges confronted by imaging genetics have been recognized and novel methodology and analytic approaches have been adopted, we believe this is an appropriate time to undertake a cooperative data integration framework to provide a global landscape of epilepsy in both clinical and research fields.

## Conclusion

This review summarized the present status of genetics and related imaging in epilepsy. Emerging knowledge of mutations points to their pathogenic variants sculpturing subtle alterations in brain microcircuits and epileptic networks during brain development. Despite advances in neuroscience and genetic research, how and what kinds of genetic variations contribute to differences in brain structure and function are unclear. Innovations in imaging genetics successfully integrate multiple lines of association data to explain the causal basis for complex traits. With the application of increasingly high-powered and multi-dimensional imaging genetics analysis, we will be able to find potential causal mutations and pathways that correlate with the imaging markers for future mechanism exploration and clinical translation.

## Author Contributions

GW was the first author of this review who referred the relevant literature and completed the first draft of this article. WW, YX, ZY, and BX modified the draft and figures. LL is the designer who is in-charge of the project and directed this review’s writing. All authors contributed to the article and approved the submitted version.

## Conflict of Interest

The authors declare that the research was conducted in the absence of any commercial or financial relationships that could be construed as a potential conflict of interest.

## Publisher’s Note

All claims expressed in this article are solely those of the authors and do not necessarily represent those of their affiliated organizations, or those of the publisher, the editors and the reviewers. Any product that may be evaluated in this article, or claim that may be made by its manufacturer, is not guaranteed or endorsed by the publisher.
